# ChemDistiller: an engine for metabolite annotation in mass spectrometry

**DOI:** 10.1093/bioinformatics/bty080

**Published:** 2018-02-12

**Authors:** Ivan Laponogov, Noureddin Sadawi, Dieter Galea, Reza Mirnezami, Kirill A Veselkov

**Affiliations:** Department of Surgery and Cancer, Faculty of Medicine, Imperial College London, London, UK; Department of Surgery and Cancer, Faculty of Medicine, Imperial College London, London, UK; Department of Surgery and Cancer, Faculty of Medicine, Imperial College London, London, UK; Department of Surgery and Cancer, Faculty of Medicine, Imperial College London, London, UK; Department of Surgery and Cancer, Faculty of Medicine, Imperial College London, London, UK

## Abstract

**Motivation:**

High-resolution mass spectrometry permits simultaneous detection of thousands of different metabolites in biological samples; however, their automated annotation still presents a challenge due to the limited number of tailored computational solutions freely available to the scientific community.

**Results:**

Here, we introduce ChemDistiller, a customizable engine that combines automated large-scale annotation of metabolites using tandem MS data with a compiled database containing tens of millions of compounds with pre-calculated ‘fingerprints’ and fragmentation patterns. Our tests using publicly and commercially available tandem MS spectra for reference compounds show retrievals rates comparable to or exceeding the ones obtainable by the current state-of-the-art solutions in the field while offering higher throughput, scalability and processing speed.

**Availability and implementation:**

Source code freely available for download at https://bitbucket.org/iAnalytica/chemdistillerpython.

**Supplementary information:**

Supplementary data are available at *Bioinformatics* online.

## 1 Introduction

Genomic, proteomic and metabolomic molecular phenotyping approaches have significantly enhanced our understanding of the biological complexities that govern the balance between health and disease. Metabolomics involves the quantitative analysis of biochemical activity within a living system brought about by gene-environment interactions. Mass spectrometry (MS) represents one of the most widely utilized approaches in metabolic phenotyping. Modern MS analytical platforms are capable of profiling thousands of metabolites with high mass accuracy. In the case of mass spectrometry imaging (MSI), it is also possible to spatially resolve the distribution of metabolites within a biological sample with up to a near-cellular or even organelle-level resolution (Duenas *et al.*, 2017). Comprehensive characterization of the metabolome promises unique insights into the biological mechanisms regulating the status (healthy versus diseased, for example) of a living organism, and these in turn can be expected to lead to significant diagnostic and therapeutic advancements. A fundamental step in any metabolic profiling experiment is the identification of detected mass spectral peaks. Although a variety of tools are currently available to assist metabolite identification [MetFrag (Ruttkies *et al.*, 2016; Wolf *et al.*, 2010), CSI:FingerID (Dührkop *et al.*, 2015), CFM-ID (Allen *et al.*, 2014) etc.], these are generally tailored to efficiently handle the annotation of small batch or individual MS datasets.

Consequently, there is a critical need for solutions to be developed with the capacity for high-volume chemical annotation from MS derived spectra. The ideal solution would operate within a computational workflow that integrates a modular annotation engine with a dedicated database of candidate compounds. The annotation engine should fulfill the following requirements: (a) provide modular design incorporating state-of-the-art metabolite annotation approaches in customizable and easily extendable fashion; (b) be scalable and permit efficient batch processing of hundreds-to-thousands of metabolite spectra, cross-referencing these against large-scale chemical compound databases; (c) support multiprocessing and permit intuitive deployment, installation, use and modification and (d) operate within an open-access platform for enhanced data transparency.

**Fig. 1.**
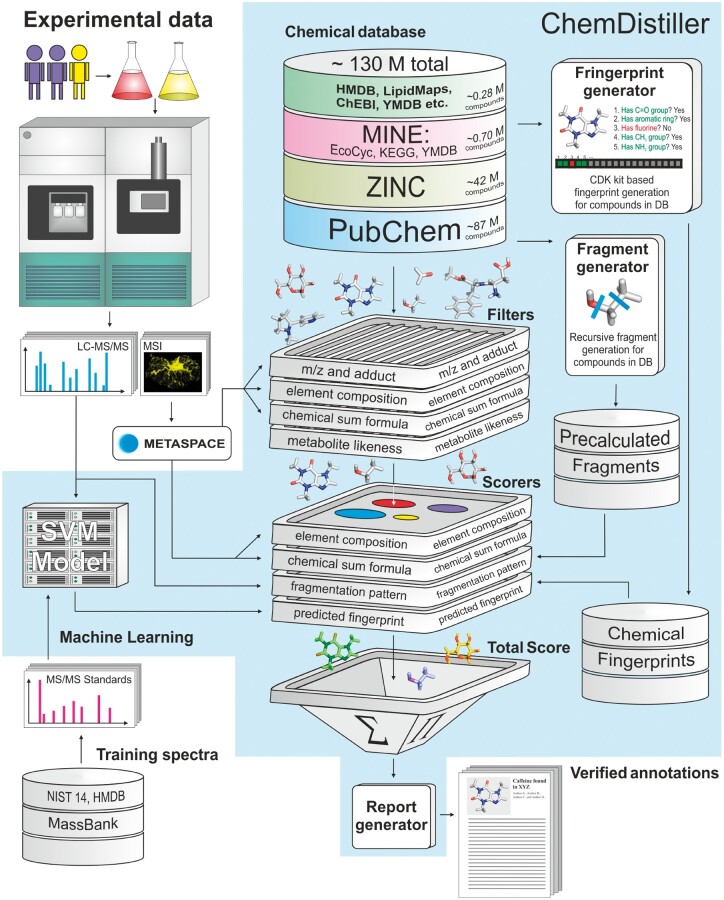
ChemDistiller. Conceptual scheme of the engine

Here, we introduce the ChemDistiller platform which has been designed specifically to address the requirements outlined above for chemical annotation of tandem-MS derived data. ChemDistiller combines a custom-developed annotation engine for retrieval, filtering and scoring of candidate molecules based on tandem-MS experimental data (Fig. 1) with the use of in-built large-scale chemical compound repositories (Fig. 1, Supplementary Table S1 and Supplementary Note S1). ChemDistiller relies on peak-picked data as the input [which can be generated by XCMS (Smith *et al.*, 2006), mzMine (Pluskal *et al.*, 2010) etc.], can benefit from additional information such as suggested formula or element composition [e.g. from SIRIUS (Bocker *et al.*, 2009) or MetaSpace (Palmer *et al.*, 2017)] and produces the lists of the best candidate compounds for the query tandem MS spectra in the hierarchical text format and as the HTML report.

## 2 Materials and methods

For large-scale data processing, it is essential to have as many biochemical properties of molecules as possible pre-computed and stored in a dedicated database in order to minimize compound annotation time. Thus, we have collected, pre-processed and compiled data from publically available chemical databases including: (a) dedicated metabolome and biological compound databases [ChEBI (Degtyarenko *et al.*, 2008), LipidMaps (Fahy *et al.*, 2007), HMDB (Wishart *et al.*, 2013) and YMDB (Jewison *et al.*, 2012)]; (b) *in silico* predicted metabolite structures [MINE database series (Jeffryes *et al.*, 2015)]; (c) pharmacologically relevant compounds [ZINC (Irwin and Shoichet, 2005)] and (d) general large-scale compound databases [PubChem (Kim, *et al.*, 2016)]. To our knowledge this covers all major publicly available and downloadable compound databases (see Supplementary Note S1 and Supplementary Table S1 for the full list of databases covered) and includes approximately 130 million chemical compounds in total. All databases have been converted into HDF5-based format for efficient data storage and access, with the following fields generated by OpenBabel/Pybel (O’Boyle *et al.*, 2011) from either SMILES (Weininger, 1988) or SDF/MDL format: InChI (Heller *et al.*, 2013), InChI key, exact mass, charge and formula (Supplementary Note S1). For each compound, chemical fingerprint and *in silico* fragmentation patterns were calculated and stored in our purpose-built HDF5-based database (Supplementary Note S1).

To perform the tandem MS spectra annotation ChemDistiller applies a series of filters to the candidate compounds stored in the compound database; the first filter step generates a list of potential candidate compounds from the user selected database(s) based on m/z value (within a specified tolerance window) derived from the MS1 peak. The user can specify, a priori, whether to use all databases available (by default) or a specific one(s) according to requirements. Filtered molecules are then subjected to a set of additional customizable filters including chemical formula, elemental composition and metabolite likeness (Fig. 1, Supplementary Note S1). Filtered candidate compounds are then ranked according to aggregated scores derived through a combination of machine-learning predicted chemical fingerprints [using an approach inspired by CSI:FingerID (Dührkop *et al.*, 2015)] and *in silico* generated compound fragmentation patterns [using MetFrag (Ruttkies *et al.*, 2016; Wolf *et al.*, 2010) and CFM-ID (Allen *et al.*, 2014; Allen *et al.*, 2015) -like approaches]. Where necessary, the modular design enables the user to extend the annotation engine with custom-developed scoring approaches.

In brief, the chemical fingerprinting approach (FingerScorer) calculates a list of chemical features for a given compound (the so-called ‘fingerprint’). This fingerprint is composed of a binary array where every feature (e.g. aromatic ring or -OH group), is set to ‘1’ if present, and ‘0’ otherwise. We united Klekota-Roth, MACCS, EState, Hybridization, PubChem, Substructure, Extended, GraphOnly and Fingerprinter fingerprint generators provided in CDK (Steinbeck *et al.*, 2006) to form a 11 416 bit long fingerprint per compound. Each fingerprint is stored in the generated databases in a compressed binary form for fast retrieval and analysis. In our application, a support vector machine-learning approach (SVM) [as implemented in libSVM software package (Chang and Lin, 2011)] was used first to train SVM models to predict molecular fingerprints from tandem MS experimental data using a set of reference ‘training’ spectra. For SVM class balance, only bits with occupancy between 0.05 and 0.95 from the entire array of fingerprint bits were considered (∼2500 bits out of 11 416 total). The obtained models are used to predict fingerprint bits for the ‘test’ compounds based on their merged tandem MS/MS spectra. Candidate compounds retrieved from chemical databases are then ranked according to the similarity of predicted versus actual molecular fingerprints using Jaccard similarity function. The best candidates are reported to the user.

The *in silico* fragmentation approach (FragScorer) directly generates fragmentation patterns that are later compared with the experimentally observed tandem MS spectra. For each molecule in the database its fragmentation pattern is calculated by recursively breaking down its chemical bonds and aggregating the resulting fragmentation masses. Up to two bonds can be broken simultaneously with the default configuration. The molecules are broken in their neutral state, unless they are inherently charged and the fragments are stored in the database for candidate retrieval. Estimation of the bond breakage rates is not used in the current implementation. For candidate molecule retrieval, the experimental tandem MS/MS spectrum is normalized so that the sum of intensities is equal to 1.0 and then matched against the pre-calculated fragmentation pattern of the candidate compounds from the database. The adduct form and isotope information are applied at this stage to the predicted fragmentation pattern to match the experimental settings (thus the same pattern from the database can be used to predict both negative and positive mode ion fragments). Fragment intensity weighted match is used as a scoring function, i.e. the score is the sum of intensities of the matched peaks. Furthermore, we found that the retrieval rates are generally improved by addition of –1/+1 hydrogen mass to the predicted peaks, possibly accounting for tautomerism (empirically established correction, 5-fold cross-validated).

When both FingerScorer and FragScorer are used together or additional scores are available from other methods, the reported scores are multiplied together by default to produce the TotalScore which is in turn used to sort candidate molecules and select the best ones. If only a single method is used, the TotalScore is set to the return value of this method. Our experimentation with different ways of combining the scores showed this approach to be the most effective and robust (data not shown), however, other methods for calculation of the combined TotalScore are easy to implement if needed. The maximal value the TotalScore can theoretically reach is 1.0 and the minimum is 0.0. In practice the values of the TotalScore are quite varied for correctly identified compounds (Supplementary Table S2), meaning that there are no absolute ‘good’ or ‘bad’ TotalScore values. However, if used relative to the maximum TotalScore value returned for the set of candidate compounds, the correct value is often close to 80–90% of the maximum value (Supplementary Table S3) both for small and large compound databases.

If multiple MS2 spectra with different collision energy are available per MS1 peak, they are merged into one for analysis and re-normalized so that the sum of intensities equals to 1.0. This is roughly equivalent to having a spectrum recorded in the ramp mode. At the moment of generation of this manuscript both FingerScorer and FragScorer did not use the collision energy information, so for the current method implementation we would recommend using either the ramp mode or several collision energies covering the range which assures ∼50% fragmentation of the original MS1 peak as a default setting. This may change in the future, however, with the new generations of ChemDistiller.

To our knowledge, the two approaches implemented (i.e. FingerScorer and FragScorer) represent the only methods that are scalable to the processing of millions of candidate compounds, unlike methods that are reliant on experimental databases limited to a smaller number of reference MS spectra.

## 3 Results and discussion

The primary objective in the development of ChemDistiller was to facilitate the annotation of tens of thousands of experimentally acquired tandem-MS spectra, and thus computational efficiency was of paramount importance. We have built upon the methodologies utilized in the existing solutions such as MetFrag (Ruttkies *et al.*, 2016; Wolf *et al.*, 2010), CFM-ID (Allen *et al.*, 2015) or CSI:FingerID (Dührkop *et al.*, 2015) (see Supplementary Figs S1 and S2 for test results and Supplementary Note S1 for details of the method implementations. See Supplementary Fig. S3 for the example of tandem MS spectrum annotation for test compounds). Our system benefits from storing calculated molecular properties and re-using these for subsequent compound annotation tasks without the need for re-calculation for each run (improvement over processing speed of MetFrag). Furthermore, our system is tailored for batch processing and does not rely on the web service availability which makes it more suitable for large scale high-throughput tandem MS spectra annotation than CFM-ID and CSI:FingerID platforms.

For fingerprint based approaches, the models were trained using a combination of commercially (NIST14) and publicly [MassBank (Horai *et al.*, 2010) and HMDB (Wishart *et al.*, 2013)] available spectral databases of pure compounds. Only high mass accuracy spectra (m/z error <20 ppm or <0.005 Da) were used. This is because our preliminary analysis indicated that retrieval methods benefit greatly from high mass accuracy data due to the reduced search space for candidate molecules, especially when used with large scale chemical databases (e.g. when used with PubChem, the mass window of 1 Da for the molecular mass of 400 Da yields 250 079 candidate molecules, while the mass window of 20 ppm yields only 3229 candidate molecules, reducing the search space ∼77.5 times). For low mass accuracy spectra (i.e. for the data from the unit mass resolution spectrometers) one would need to use small tailored chemical databases (e.g. HMDB) in order to achieve sufficiently high retrieval rates. Redundant compound repetitions across multiple databases were filtered out based on their InChI identifiers (Heller *et al.*, 2013), leaving a final set of 6297 compounds with their corresponding tandem MS spectra. These data were split at random into training and test batches (80%:20%). Training and optimization of modeling parameters was performed on the training batch (80%) using nested 5-fold cross validation with the remaining batch (20%) being reserved for testing to minimize bias. The same training dataset was used to train CFM-ID and CSI:FingerID models (Supplementary Notes 3 and 4) and the same test dataset was employed throughout in order to compare our approach to the current state-of-the-art (MetFrag, CFM-ID and CSI:FingerID, Supplementary Notes 2–4). Retrieval rates were separately acquired for small (combination of HMDB, ChEBI, MassBank and NIST14) and large (combination of PubChem, HMDB, ChEBI, MassBank and NIST14) databases to estimate the performance of our methods and their combinations as well as compare them to the methods implemented in MetFrag, CFM-ID and CSI:FingerID under different conditions. In the case of the small database (HMDB, ChEBI, MassBank and NIST14; in combination totaling ∼130 thousand compounds), 24 candidates were on average retrieved per single MS1 peak, of which there were 1153 submitted for annotation (862 in positive ion mode and 291 in negative ion mode). For the large database (PubChem, HMDB, ChEBI, MassBank and NIST14; in combination totaling ∼87 million compounds), ∼10 000 candidates were on average retrieved per single MS1 peak for the same set of peaks submitted for annotation. The performance results for all modeling approaches for the test batch are summarized in Table 1 and Supplementary Figures S1 and S2, where 20 ppm or greater mass accuracy has been assumed throughout for database compound retrieval.

**Table 1.** Retrieval statistics (per cent)

	Positive mode			Negative mode		
	Baseline performance	+Element filter	+Known formula	Baseline performance	+Element filter	+Known formula
Small database (HMDB, MassBank, ChEBI, NIST14) Correct in **TOP 1**					
FingerScorer	37.5 ± 3.5	44.5 ± 3.5 (+7.0)	46.0 ± 3.0 (+8.5)	32.5 ± 4.5	37.5 ± 4.5 (+5.0)	38.5 ± 4.5 (+6.0)
FragScorer	50.0 ± 2.0	54.0 ± 2.0 (+4.0)	56.0 ± 2.0 (+6.0)	44.0 ± 3.0	45.5 ± 3.5 (+1.5)	47.0 ± 4.0 (+3.0)
FingerScorer&FragScorer	49.0 ± 2.0	52.0 ± 2.0 (+3.0)	53.0 ± 2.0 (+4.0)	42.5 ± 3.5	45.0 ± 3.0 (+2.5)	46.0 ± 3.0 (+3.5)
MetFrag	51.7 ± 0.0	51.7 ± 0.0 (+0.0)	51.7 ± 0.0 (+0.0)	45.0 ± 0.0	45.0 ± 0.0 (+0.0)	45.0 ± 0.0 (+0.0)
CFM-ID^a^	41.0 ± 4.0	47.5 ± 3.5 (+6.5)	48.5 ± 2.5 (+7.5)	36.5 ± 4.5	41.5 ± 3.5 (+5.0)	42.5 ± 3.5 (+6.0)
CSI:FingerID	N/A	42.5 ± 2.5	45.0 ± 3.0 (+2.5)	N/A	38.0 ± 5.0	39.0 ± 5.0 (+1.0)
Small database (HMDB, MassBank, ChEBI, NIST) Correct in **TOP 5**						
FingerScorer	80.0 ± 3.0	83.0 ± 3.0 (+3.0)	83.5 ± 2.5 (+3.5)	71.5 ± 5.5	74.5 ± 5.5 (+3.0)	75.0 ± 5.0 (+3.5)
FragScorer	82.5 ± 2.5	84.0 ± 2.0 (+1.5)	84.5 ± 2.5 (+2.0)	76.5 ± 4.5	77.5 ± 4.5 (+1.0)	77.5 ± 4.5 (+1.0)
FingerScorer&FragScorer	86.0 ± 2.0	87.0 ± 2.0 (+1.0)	87.0 ± 2.0 (+1.0)	78.0 ± 4.0	80.0 ± 4.0 (+2.0)	80.0 ± 4.0 (+2.0)
MetFrag	86.5 ± 0.0	88.6 ± 0.0 (+2.1)	89.1 ± 0.0 (+2.6)	83.5 ± 0.0	85.6 ± 0.0 (+2.1)	85.6 ± 0.0 (+2.1)
CFM-ID^a^	76.5 ± 3.5	80.5 ± 3.5 (+4.0)	82.0 ± 2.0 (+5.5)	68.0 ± 7.0	74.0 ± 2.0 (+6.0)	74.0 ± 2.0 (+6.0)
CSI:FingerID	N/A	76.0 ± 3.0	76.5 ± 2.5 (+0.5)	N/A	69.0 ± 5.0	69.5 ± 4.5 (+0.5)
Large database (PubChem, HMDB, MassBank, ChEBI) Correct in **TOP 20**						
FingerScorer	48.5 ± 4.5	52.0 ± 5.0 (+3.5)	56.5 ± 5.5 (+8.0)	47.0 ± 7.0	52.5 ± 8.5 (+5.5)	56.0 ± 9.0 (+9.0)
FragScorer	26.5 ± 1.5	34.5 ± 1.5 (+8.0)	41.5 ± 1.5 (+15.0)	36.5 ± 2.5	45.0 ± 4.0 (+8.5)	45.5 ± 3.5 (+9.0)
FingerScorer&FragScorer	45.0 ± 3.0	49.5 ± 3.5 (+4.5)	52.5 ± 3.5 (+7.5)	50.5 ± 7.5	56.0 ± 8.0 (+5.5)	58.5 ± 8.5 (+8.0)
MetFrag	32.8 ± 0.0	41.4 ± 0.0 (+8.6)	50.0 ± 0.0 (+17.2)	41.2 ± 0.0	48.5 ± 0.0 (+7.3)	50.2 ± 0.0 (+9.0)
CFM-ID^a^	23.0 ± 1.0	31.5 ± 1.5 (+8.5)	37.5 ± 1.5 (+14.6)	30.0 ± 0.0	34.5 ± 0.5 (+4.5)	38.0 ± 1.0 (+8.0)
CSI:FingerID	N/A	34.5 ± 4.5	38.0 ± 5.0 (+3.5)	N/A	41.5 ± 6.5	43.5 ± 6.5 (+2.0)

a For CFM-ID the best retrieval rate is reported for all scoring functions used, however the best performing scoring function was not consistent between different tests (Supplementary Note S3). Improvement relative to baseline performance is shown in brackets.

### 3.1 ChemDistiller performance

Overall, our methodology achieved up to 37.5%/32.5% (here and further: positive/negative experimental acquisition mode) correct annotations in top 1 (i.e. the correct annotation will be among the first top *n* candidates in 37.5%/32.5% of the test cases with *n* being equal to 1 in this case) for the small database with FingerScorer and up to 50%/44% correct annotations using FragScorer. The combination of both approaches performed slightly less well for the top 1 rank (49%/42.5%) compared to FragScorer on its own. However, for the correct annotation in top 5 using the small database, the combination of the two was shown to outperform any one approach used in isolation (combined 86%/78% versus individual 80%/71.5% and 82.5%/76.5% for FingerScorer and FragScorer, respectively). For the large database, FingerScorer significantly outperformed FragScorer with the retrieval rates for the top 20 being 48.5%/47% versus 26.5%/36.5% for FingerScorer and FragScorer, respectively. The combination of the two scorers performed slightly less efficiently compared to the FingerScorer on its own for positive mode (45% versus 48.5%), however in negative mode the combined approach was found to be superior (50.5% for the combination, 47.0% for FingerScorer). Overall the combination of the two scorers either outperforms the individual scorers, or closely follows the best individual scorer (i.e. FragScorer for the small, metabolite-targeting database and FingerScorer for the large general database). As such the combination of the two scorers can be recommended in circumstances where overall robustness is more critical than the top possible performance.

Furthermore, addition of the elemental composition information or known chemical formula [for example, determined by SIRIUS (Bocker *et al.*, 2009) or MetaSpace (Palmer *et al.*, 2017) engine] can increase the retrieval rates further by 1–8.5% for the small and 3.5–17.2% for the large databases, respectively. Interestingly, the comparatively weaker scorers benefit generally more from the elemental composition and known chemical formula information; this is best exemplified in the case of FragScorer, where retrieval rates for the top 20 candidates from the large chemical database was seen to increase from 26.5% to 41.5% (Table 1).

All retrieval rate tests were performed using our standalone workstation (8 core Intel ^®^ Xeon ^®^ E5-2630 v3 @2.4 GHz, 64 Gb RAM). The number of available CPU cores was limited to 6 for worker threads + 1 for the main thread for these tests. Processing of 1153 test spectra took from 2 min using FragScorer on its own (30 min with FragScorer and FingerScorer combined) and a small database (HMDB, MassBank, NIST14, ChEBI; in combination totaling ∼130 thousand candidate compounds) to 4–4.5 h using both FragScorer and FingerScorer and a large database (PubChem, HMDB, ChEBI, MassBank and NIST14; in combination totaling ∼87 million candidate compounds). The memory footprint registered was of ∼4 Gb for the main thread and ∼400 Mb per worker thread. These results show that tandem MS spectra (each corresponding to the individual compound of interest) can be annotated at a rate of at least several thousand spectra per day using an ordinary desk-top PC (4–6 cores, 16 Gb RAM recommended, ∼80 Gb HDD free space for the full set of databases and SVMs) when employing the combination of both FingerScorer and FragScorer and the large database of ∼87 million candidate compounds (FingerScorer was used with the radial kernel SVM for better prediction at the expense of the longer running times). If smaller tailored databases are used, the number of spectra annotated per day can easily reach 50 000–1 000 000 depending upon the selection of the scoring methods. Further speed-ups can be achieved by using SSD disks for the database storage or deployment of ChemDistiller onto HPC cluster.

### 3.2 ChemDistiller compared to the state-of-the-art solutions available in the field (i.e. MetFrag, CFM-ID and CSI:FingerID)

Compared with other state-of-the-art solutions in the field, we have found that under the same conditions MetFrag can slightly outperform our best scorer for the top 1 position in the case of the small database (51.7%/45% versus 50%/44%) or the combination of our scorers for the top 5 (86.5%/83.5% versus 86%/78%). However, when applied to the large database our methods performed noticeably better (32.8%/41.2% versus 48.5%/47% versus 45%/50.5% for MetFrag, FingerScorer and the combination of the FingerScorer and FragScorer, respectively, see Table 1). Furthermore, thanks to the stored pre-calculated fragmentation patterns in the database, our method performs ∼6–8 times faster than the current implementation of MetFrag where fragmentation patterns are calculated each time ‘on the fly’. The noticeable discrepancy between retrieval rates for positive and negative mode data is likely attributed to the differences in (a) the size of the test/training data and (b) the molecular weight and molecular type distribution differences between the datasets of the two experimental acquisition modes. Overall, in our experience, the command line version of MetFrag (Ruttkies *et al.*, 2016) proved to be the most simple to use and robust among the available state-of-the-art solutions in the field. MetFrag is very efficient at *in silico* fragmentation based retrieval. It would be a very useful addition to the scoring methods presented herein, if the possibility of pre-calculation and storing of the predicted fragmentation patterns from MetFrag was realized.

When comparing our engine to CFM-ID (Allen *et al.*, 2014; Allen *et al.*, 2015) we had to focus on the single energy method implemented in CFM-ID (Supplementary Note S3) as the multiple energy method was found to be too computationally expensive for the entire training dataset with no significant improvement in the performance relative to the single energy one. To our surprise, in all our tests our methods managed to provide better annotation scores than CFM-ID (improvement ranged from ∼4% to ∼20% depending upon the acquisition mode, used method and the database size, see Table 1).

For CSI:FingerID, we used SIRIUS to generate fragmentation trees and then train and optimize parameters of the kernels used by the FingerID python script (Supplementary Note S4). The retrieval rates obtained for CSI:FingerID were close to the ones observed for our methods, however our combined methods (FingerScorer and FragScorer) outperformed CSI:FingerID by a small margin in all the tests (improvement ranged from ∼8% to ∼15% depending upon the acquisition mode and the database size, see Table 1).

### 3.3 Results overlap between different methods

Interestingly, for all methods the returned results did not overlap 100% (Supplementary Table S5) with the overlap percentages varying from 74% to 93% for small molecular databases (HMDB, NIST14, MassBank, ChEBI) and from 27% to 78% for large molecular databases (PubChem, HMDB, MassBank, ChEBI) depending on the combinations of methods and filters used. This means that the success of the annotation is both method and query molecule dependent, allowing us to theorize that for different classes of metabolites selective application of the methods could prove to be a more effective strategy than a direct combination of available methods to generate a single TotalScore. However, it remains to be seen if it is possible to determine the best method to use based on the spectrum of the query compound in a situation when the class of this compound is not known a priori. Though answering this question falls outside of the scope of this paper, we anticipate it could open up a new area of research in the field of annotation of the tandem MS spectra of metabolites.

### 3.4 Metabolite likeness filter

In an attempt to further improve the retrieval performance of our solution, we implemented machine learning based classification of candidate molecules into metabolites and non-metabolites according to their ‘metabolite-likeness’ using an approach similar to that proposed previously by J.E. Peironecely *et al.* (Peironcely *et al.*, 2011). This approach is expected to help reduce the search space for large databases by focusing only on compounds which are likely to originate from living organisms. HMDB (Wishart *et al.*, 2013), MassBank (Horai *et al.*, 2010), ChEBI (Degtyarenko *et al.*, 2008), PlantCyc (Zhang *et al.*, 2010), ECMDB (Guo *et al.*, 2013), YMDB (Jewison *et al.*, 2012), BMDB (Bovine Metabolome Database) and LipidMaps (Fahy *et al.*, 2007) were assumed to be the databases of metabolites, while the selection of compounds from ZINC (Irwin and Shoichet, 2005) (Supplementary Note S5) were assumed to be non-metabolites. We used pre-calculated fingerprints to train a two-class SVM, achieving ∼95% accuracy with 5-fold cross-validation. The model was applied to calculate metabolite likeness for all compounds in our collection of databases and the appropriate filter was added to the annotation pipeline. In general metabolite databases such as HMDB (Wishart *et al.*, 2013), LipidMaps (Fahy *et al.*, 2007) and YMDB (Jewison *et al.*, 2012) were predicted to contain over 90%, while PubChem (Kim *et al.*, 2016) was estimated to have ∼35% and ZINC (Irwin and Shoichet, 2005)–∼20% metabolite like entries (see Supplementary Table S4 for the full list of predicted percentages of metabolite like entries). Approximately 79% of our NIST/MassBank (Horai *et al.*, 2010) reference compounds were classified as metabolites and the subsequent analysis was performed using these metabolite-like compounds only to estimate the improvement in retrieval with the addition of this filter. PubChem (Kim *et al.*, 2016) was selected as a representative large database. We observed a decrease in the average number of candidates per annotation from ∼10 k down to ∼4 k with the metabolite likeness filter applied and further down to 1 k with the known formula assumed. However, the improvements observed in retrieval rates were comparatively more modest (Supplementary Fig. S6) adding 3–4% improvement on average, compared to 6–9% gained by the addition of known formula. This can be explained by the majority of filtering power being provided by the tandem MS based methods in this case.

In particular, machine learning based approach of FingerScorer seem to dominate in terms of performance when applied to large scale compound databases. This potentially could be explained by the initial bias of the training set of molecules towards the ‘biologically relevant’ ones (e.g. metabolites, drugs) as those are generally of a higher interest to the scientific community and the databases of the reference compound tandem MS spectra would target those first. One can see it as ‘metabolite likeness’ filtering being already partially realized as part of the FingerScorer method and when applied to the large databases, ‘biologically relevant’ molecules are more likely to be picked first than the ‘biologically irrelevant’ ones. This also explains why the search against small databases of biologically relevant compounds does not benefit as much from machine learning approach–the databases are already focused on biological compounds and thus the benefit of the ‘biological relevance’ bias is negated. The bias towards ‘biological relevance’ of the FingerScorer is not possible to overcome without additional training datasets being available which would focus on non-‘biologically relevant’ compounds. Thus, our recommendation is to use both FingerScorer and FragScorer together in the situation where the ‘biological relevance’ of the compound in question is not yet determined. FragScorer is relying on a set of more general rules of fragmentation and is not biased towards ‘biologically relevant’ compounds.

In the current version of ChemDistiller, the metabolite likeness filter is not enabled by default in order to avoid exclusion of potentially important non-metabolites (e.g. synthetic drugs) and should be switched on by the user where needed.

## 4 Conclusion

In conclusion, ChemDistiller is comparable to the state-of-the-art tools available for metabolite annotation using tandem MS data while at the same time offering customizability, speed and high-throughput batch processing. It supports multi-processing and multiple operating systems. With its core written in Python, it is easy to install, use and further develop/expand. It has minimal library dependencies and modular architecture with standardized interfaces for filter and scorer classes.

In the near future, we plan to develop an independent web interface and a server-based solution for individual and batch processing of tandem MS data. Additional filters and scorers will be developed and deployed as new experimental data becomes available and downloadable databases and SVM models are updated accordingly, with particular focus on the collision cross-section energy as an alternative to LC (liquid chromatography) retention time prediction.

## Supplementary Material

Supplementary Data
