# Supplementary material for: ChemDistiller: an engine for metabolite annotation in mass spectrometry
Source: Bioinformatics. 2018 Feb 12;34(12):2096–102. doi: 10.1093/bioinformatics/bty080 (PMC9881669; doi:10.1093/bioinformatics/bty080)
Supplement: Supplementary Data [file bioinformatics_34_12_2096_s2.doc]

**Supplementary Data**

ChemDistiller: an integrated engine for metabolite annotation in mass spectrometry

Ivan Laponogov[[1]](#footnote-2), Noureddin Sadawi^1^, Dieter Galea^1^, Reza Mirnezami & Kirill A. Veselkov^1*^

**Supplementary Note 1.** ChemDistiller development

20 publicly available compound databases have been downloaded in their native formats and each converted to the standardized format for ChemDistiller using tailored importing scripts. OpenBabel/Pybel (O'Boyle, et al., 2011) was used to generate exact mass, charge, InChI (Heller, et al., 2013) and standardized SMILES (Weininger, 1988) for the imported compounds. InChI keys were generated using our implementation of InChI key generator from InChI-s, which is identical in functionality to the original generator from InChI library, but instead implemented in Python to eliminate the C++ InChI library dependency. Compounds were separated into three categories: negatively, positively and neutrally charged. In each category compounds were sorted according to their mass (in case of neutral molecules) or mass-to-charge ratios (in case of charged ones). This allows for rapid querying of compounds from the database. Databases are stored in HDF5 format with gzip compression enabled, which allows for fast data retrieval, compact storage and modular expandability. **Table S1** lists the currently available databases for ChemDistiller.

**Supplementary Table 1**. Chemical compound databases with fingerprints and fragmentation patterns predicted

| Database | | Description | Compound Count | Source URL |
| --- | --- | --- | --- | --- |
| BMDB |  | Bovine metabolome | 7,834 | <http://www.cowmetdb.ca/cgi-bin/browse.cgi> |
| ChEBI |  | Chemical Entities of Biological Interest | 81,753 | <https://www.ebi.ac.uk/chebi/> |
| DrugBank |  | Drugs | 7,013 | <https://www.drugbank.ca/> |
| ECMDB |  | *E. coli* metabolome | 3,730 | <http://ecmdb.ca/> |
| EMolecules |  | Purchasable screening compounds | 7,938,551 | <https://www.emolecules.com/> |
| FooDB |  | Food constituents | 22,763 | <http://foodb.ca/> |
| HMDB |  | Human metabolome | 41,758 | <http://www.hmdb.ca/> |
| LipidMaps |  | Lipids | 40,228 | <http://www.lipidmaps.org/> |
| MassBank |  | HQ Mass spectra | 11,865 | <http://www.massbank.jp/?lang=en> |
|  | \EcoCycMINE | Metabolic *in silico* network expansion databases | 52,864 | [http://minedatabase.mcs.anl.gov/#/home](http://minedatabase.mcs.anl.gov/#/home) |
| MINE | \KEGGMINE |  | 556,666 |  |
|  | \YMDBMINE |  | 98,539 |  |
| Phenol | \Compounds | Polyphenol contents in food | 489 | <http://phenol-explorer.eu/> |
| Explorer | \Metabolites |  | 366 |  |
| PlantCyc |  | Plant metabolome | 50,931 | <http://www.plantcyc.org/> |
| PubChem |  | Compound database | 86,963,867 | <https://pubchem.ncbi.nlm.nih.gov/> |
| SMPDB |  | Small molecule pathway database | 4,297 | <http://smpdb.ca/> |
| T3DB |  | Toxins | 3,339 | <http://www.t3db.ca/> |
| UNPD |  | Universal natural products database | 228,789 | <http://pkuxxj.pku.edu.cn/UNPD/download.php> |
| YMDB |  | Yeast metabolome | 1,997 | <http://www.ymdb.ca/> |
| Zinc |  | Commercially-available compounds for virtual screening | 42,241,167 | <http://zinc.docking.org/> |

New additional databases can be generated from a list of SMILES with corresponding ID numbers and then copied to the specified database folder in ChemDistiller. They will be automatically recognized the next time ChemDistiller is started. ChemDistiller contains a number of importing and conversion scripts to recalculate the needed parameters for the database population.

For each compound in the compiled database fingerprint and fragmentation patterns were calculated and stored for rapid retrieval by the FingerScorer and FragScorer methods respectively. Fingerprints were calculated by our Java-based script which relies on the CDK (Steinbeck, et al., 2006) library. It combines Klekota-Roth, MACCS, EState, Hybridization, PubChem, Substructure, Extended, GraphOnly and Fingerprinter fingerprint generators provided by CDK outputting a 11416 bit long fingerprint per compound. Fragmentation pattern is predicted by our python implementation of the recursive bond breaking algorithm. Up to two bonds can be broken at a time. Two bond fragmentation limit was found to be the optimal at this stage both in terms of speed of generation and retrieval rates. No intensity prediction is attempted and peaks are stored as the lists of their m/z values only.

For testing and SVM training we collected and compiled a set of tandem MS spectra of known compounds from NIST14, HMDB and MassBank . All spectra were normalized to their sums of intensities. Reference compounds were grouped by their "short" InChI (i.e. stereoisomer information layer of InChI was ignored) treating stereo isomers as one compound. It was essential to ensure that training and test sets do not contain the same compounds to reduce the possibility of bias. Our initial analysis showed that retrieval rates drop sharply for compounds with spectra recorded with mass accuracy of less than 0.05-0.1 Da. The optimal retrieval rates were achieved for compounds with estimated mass accuracy of 0.01-0.005 Da. Estimation was done based on the level of rounding of m/z values as more detailed experimental information is unavailable for many spectra. Spectra with lower mass accuracy were removed from further consideration leaving 6297 unique compounds. Unique compounds were split randomly into 20%/80% test/training sets, respectively. Model training and parameter optimization were performed on the training set with internal 5-fold cross-validation for all the methods. Testing was performed on the test set in all cases (1153 unique compounds with corresponding tandem MS spectra, 862 in positive mode and 291 in negative mode). Only [M+H]^+^ and [M-H]^-^ adducts were considered due to the insufficient amount of reference spectra containing other adducts. Multiple energy spectra were merged together after each individual spectrum was normalized to its sum of peak intensities for our FingerScorer and FragScorer.

FingerScorer method uses SVM models to predict fingerprints from binned tandem MS spectra. Only fingerprint bits which are set "on" in 5%-95% compounds are used leaving ~2.5k features on average to be considered out of original 11416. SVM models were trained using both linear and radial kernels and 5‑fold internal cross-validation was employed for parameter optimization in LibSVM(Chang and Lin, 2011). Results presented in this paper are based on the radial kernel as it provided higher retrieval rates, although, at the expense of longer running times. Jaccard scoring was found to be the most efficient for compound retrieval using FingerScorer and thus is implemented by default.

In FragScorer, the fragmentation pattern is matched with the experimentally observed tandem MS peaks within 20 ppm tolerance and weighted with the observed peak intensities. Peaks within +1/-1 hydrogen mass are also tested for matching, accounting for potential tautomerism and providing further increase in retrieval rates (verified using 5-fold cross-validation).

For testing purposes if several candidates in the results list have total scores identical to the correct one, the correct position is assumed to be the average between the first candidate position and the last candidate position in the list with this score as theoretically the correct candidate can occur anywhere in this list of identically scored compounds. Results summary is presented in **Table 1** of the main text and in **Figures S1**&**S2**. **Figure S3** shows two examples of the tandem MS spectra annotation for test compounds in positive and negative acquisition modes.

**
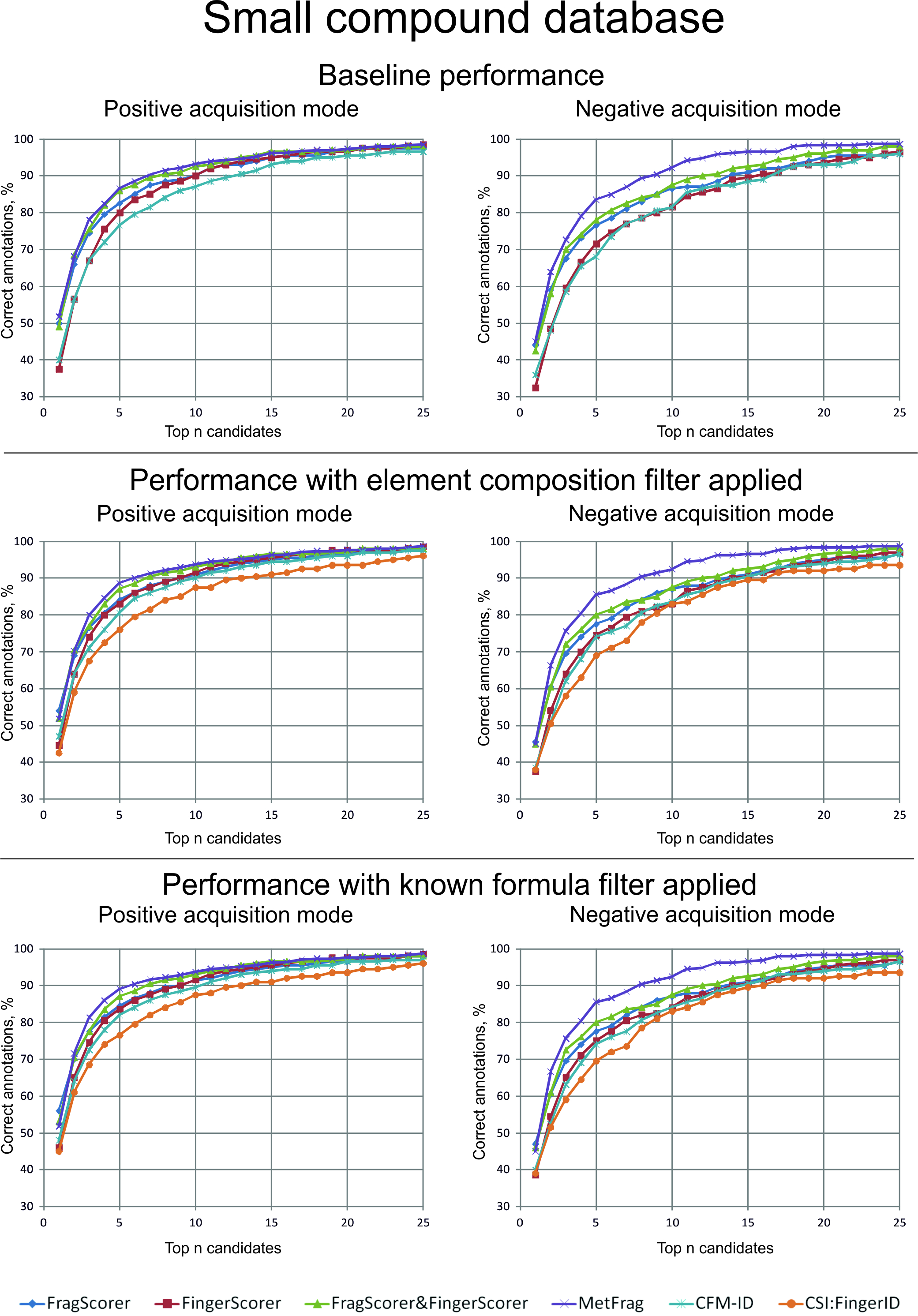
**

**Supplementary Figure 1.** Results of statistical testing of retrieval performance of ChemDistiller using small molecular database (HMDB (Wishart, et al., 2013), ChEBI (Degtyarenko, et al., 2008), MassBank (Horai, et al., 2010) and NIST14 combined) for different filters and scorers in comparison to other state-of-the-art software solutions (CFM-ID (Allen, et al., 2014), MetFrag (Ruttkies, et al., 2016), CSI:FingerID (Dührkop, et al., 2015)).

**
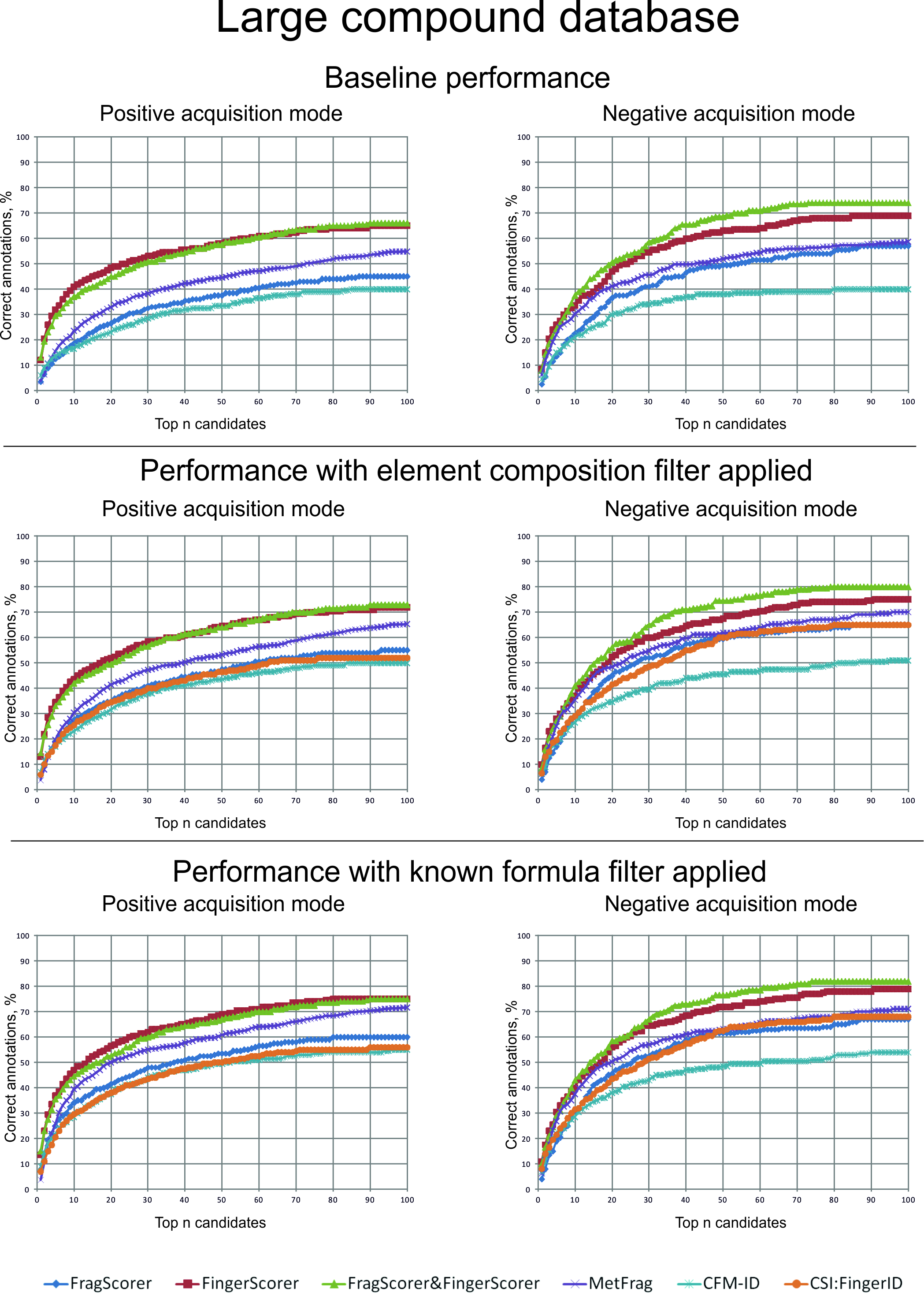
**

**Supplementary Figure 2.** Results of statistical testing of retrieval performance of ChemDistiller using large molecular database (PubChem (Kim, et al., 2016), HMDB (Wishart, et al., 2013), ChEBI (Degtyarenko, et al., 2008) and MassBank (Horai, et al., 2010) combined) for different filters and scorers in comparison to other state-of-the-art software solutions (CFM-ID (Allen, et al., 2014), MetFrag (Ruttkies, et al., 2016), CSI:FingerID (Dührkop, et al., 2015)).

**
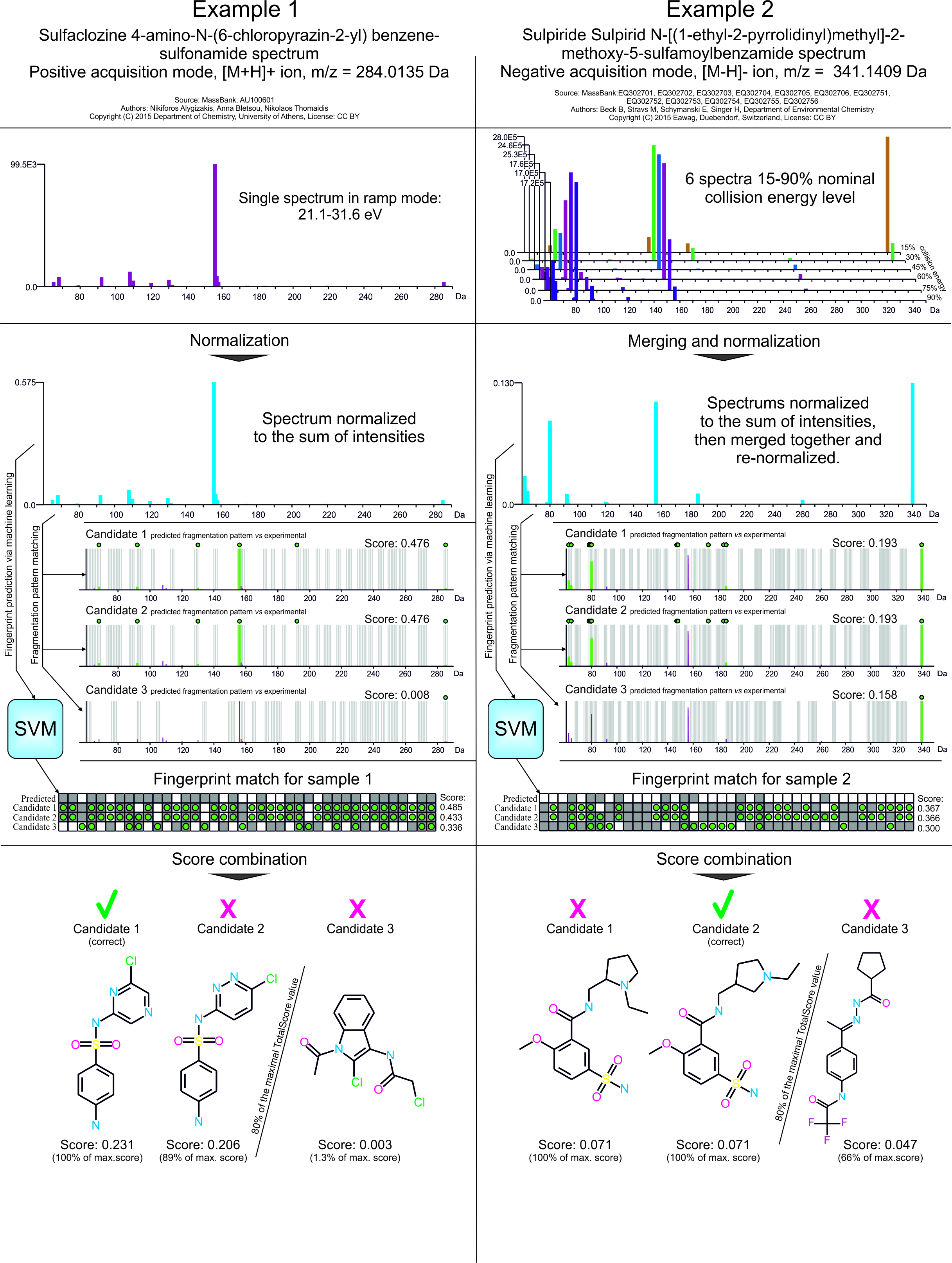
Supplementary Figure 3.** Two examples of the tandem MS spectra annotation using test compound spectra from MassBank and a small compound database (HMDB, MassBank, NIST14, ChEBI). Successful matches for the predicted fingerprints and fragments (within +/- 20 ppm) are indicated by green circles. In example 1 the correct annotation was found in the top 1 position, followed closely by the second candidate with a very similar structure and scores. In example 2 the correct candidate was returned second with almost identical scores with the first candidate (a very close isomeric form of the correct candidate molecule). In both examples the first two solutions are close in score values due to a very high degree of structural similarity and the third candidate produced scores of less than 80% of the maximum score and was clearly different from the two top solutions.

**Supplementary Table 2**. Statistics for the absolute values of TotalScore for the correct candidate molecule returned by ChemDistiller

|  | Small database  (HMDB, MassBank, ChEBI, NIST14) | | | Large database  (PubChem, HMDB, MassBank, ChEBI) | | |
| --- | --- | --- | --- | --- | --- | --- |
| Scorer: | FingerScorer | FragScorer | FingerScorer & FragScorer | FingerScorer | FragScorer | FingerScorer & FragScorer |
| Min | 0.04 | 0.00 | 0.00 | 0.10 | 0.00 | 0.00 |
| Max | 0.95 | 0.64 | 0.42 | 0.95 | 0.59 | 0.42 |
| Median | 0.53 | 0.23 | 0.09 | 0.63 | 0.23 | 0.12 |
| Mean | 0.53 | 0.23 | 0.11 | 0.63 | 0.24 | 0.13 |
| Standard Deviation | 0.23 | 0.12 | 0.07 | 0.18 | 0.11 | 0.08 |

**Supplementary Table 3**. Statistics for the values of TotalScore for the correct candidate compound relative to the values of the TotalScore for the highest rated candidate compound (i.e. TotalScore/**max**(TotalScore))

|  | Small database  (HMDB, MassBank, ChEBI, NIST14) | | | Large database  (PubChem, HMDB, MassBank, ChEBI) | | |
| --- | --- | --- | --- | --- | --- | --- |
| Scorer: | FingerScorer | FragScorer | FingerScorer & FragScorer | FingerScorer | FragScorer | FingerScorer & FragScorer |
| Min | 15% | 0% | 0% | 34% | 0% | 0% |
| Max | 100% | 100% | 100% | 100% | 100% | 100% |
| Median | 95% | 100% | 97% | 92% | 84% | 85% |
| Mean | 90% | 89% | 87% | 90% | 81% | 80% |
| Standard Deviation | 13% | 20% | 20% | 9% | 18% | 19% |

**Supplementary Note 2.** MetFrag performance testing

Command line version of MetFrag (Ruttkies, et al., 2016) was downloaded from: http://c‑ruttkies.github.io/MetFrag/.

Compiled databases were used to extract InChI-s and exact masses for candidate molecules within +/- 20 ppm of the masses of test compounds to form prepared local databases for MetFrag to search against. This way we could compare the performance of MetFrag method with our scorers directly instead of relying on the on-line querying of PubChem for candidate compounds. MetFrag was run on a single core of our main workstation (8 core Intel ® Xeon ® E5-2630 v3 @2.4 GHz, 64 Gb RAM) under Windows 7. The total running time was 8 days. Produced results were compiled using custom developed Python scripts.

**Supplementary Note 3.** CFM-ID performance testing

CFM-ID (Allen, et al., 2014) source code was downloaded and compiled under Linux both for local workstation and HPC cluster use according to the instructions from:

https://sourceforge.net/p/cfm‑id/wiki/Home/.

Default parameters and settings were used as recommended from: https://sourceforge.net/p/cfm‑id/code/HEAD/tree/supplementary_material/trained_models/.

CFM‑ID requires strictly three energy spectra to operate in the order of increasing collision energy. To satisfy this requirement reference tandem MS spectra were processed as follows:

a) All spectra had their weighted mean m/z calculated using peak intensities as weights to assess the relative collision energy and sort the spectra according to its increase. This was necessary as not all records contain clear indication of the collision energy settings used in the experiment.

b) All spectra were individually normalized to their respective sums of peak intensities.

c) If one spectrum was available for the test compound, it was used thrice; if two - the third middle spectrum was calculated as a sum of the two spectra divided by 2; if three or more spectra were available - spectra were grouped and merged in three energy groups via nearest neighbour approach. Should the middle group happen to be empty - it was recreated as in case of two spectra available.

Training of both single energy (SE) and three energy (CE) models was attempted for both negative and positive modes. CE models showed inferior performance compared to the SE models at the beginning of the learning curve with little indication of improvement with the increase of the fraction of the training set used. At the same time computational time increased beyond 2 weeks per try and thus CE models were not considered further. SE models remained manageable, although showing small improvement with the increase of the training set (see **Figure S4**). All scoring metrics used in the CFM-ID publication were tested (i.e. Jaccard, dot product, weighted precision, weighted recall, precision and recall) for individual predicted spectra and for the combination of the three energy spectra showing similar behaviour.


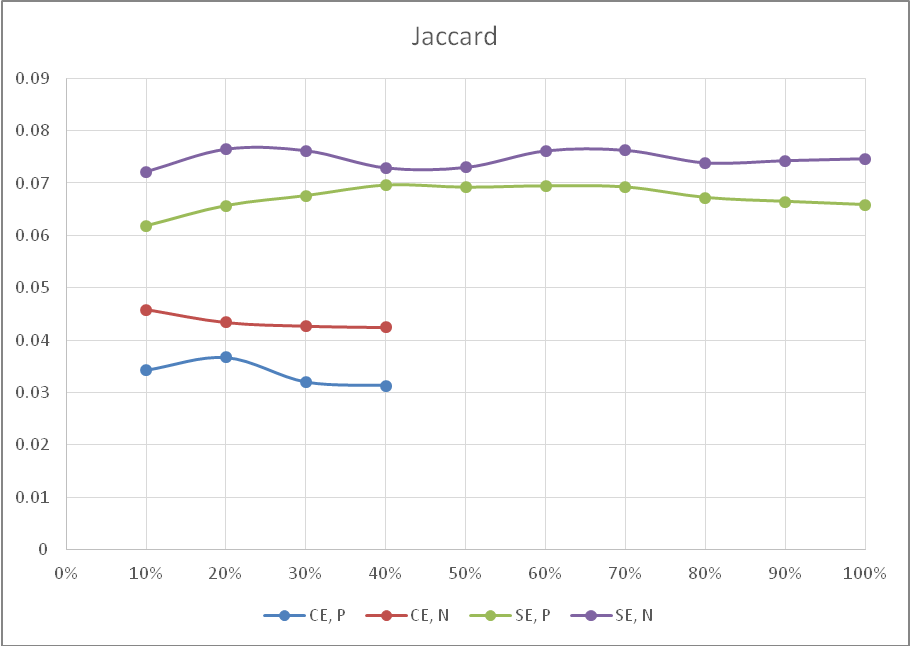


**Supplementary Figure 4**. Learning curve for CE and SE models of CFM-ID. Positive mode indicated as P, negative as N. Horizontal axis indicates the size of the training set used (%), vertical axis indicates correlation between the experimental and predicted spectra in the test set.

Trained SE models from the full training set were used to calculate predicted three energy spectra for the candidate compounds within +/- 20 ppm of the masses of test compounds. This computation for 11 million candidate compounds was performed using HPC cluster and required over 2 months in total with a number of unexplained crushes and restarts for several compounds. These predicted spectra were ported into our database collection and CFM-ID based scorer was developed. Different scoring functions were tested to find the best one for retrieval, i.e. Jaccard, dot product, weighted precision, weighted recall, precision and recall. Scores from multiple energy spectra were combined using multiplication and Euclidian summation. Merging three energy spectra into one prior to scoring was also tried. Finally, matching low, middle and high energy spectra was either attempted 1:1 (CFM) or using the closest spectra match according to a weighted mean of m/z values of peaks (CFM2) with intensities of the peaks used as weights (i.e. in attempt to match spectra with the closest fragmentation levels/collision energy). Interestingly, in most of the cases simple matching 1:1 of low, medium and high energy spectra worked better than tailored matching of the closest spectra in terms of mean weighted m/z. Also, to our surprise, none of the scoring methods managed to exceed our combined scores despite CFM-ID using more advanced algorithms (see **Figure S5**). Furthermore, the best scoring method could not be detected consistently and systematically.


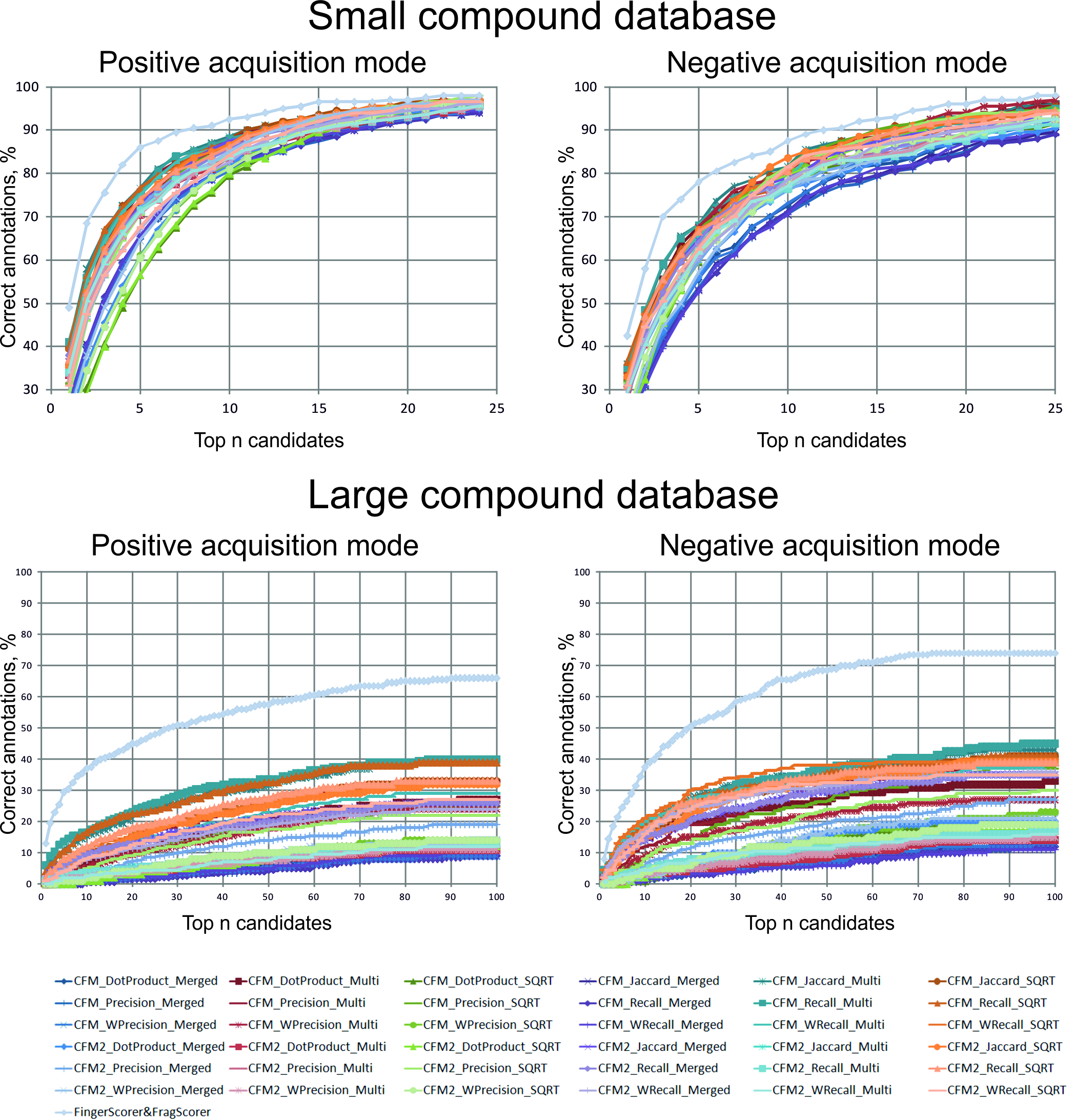


**Supplementary Figure 5.** Retrieval rates using different scoring methods for CFM-ID predicted spectra for large compound database. Retrieval rates for the combination of FingerScorer and FragScorer combined are shown for the reference.

**Supplementary Note 4**. CSI:FingerID testing

SIRIUS(Bocker, et al., 2009) was downloaded from:

https://bio.informatik.uni-jena.de/software/sirius/

and used to generate fragmentation trees to be used by FingerID (Dührkop, et al., 2015; Shen, et al., 2014). For training set the known formula was provided for fragmentation tree generation. For the test set it was assumed that the known formula should be established by SIRIUS with several plausible solutions to be considered where appropriate. However, a large number of possible atomic formulas for compounds with higher molecular weight made it computationally unfeasible to predict all possible formulas and fragmentation trees for the test set. As a result prediction had to be restricted to the known elemental compositions, thus no "ground" level performance was estimated for the situation when no prior information about the molecule was available (see **Table 1** in the main text). Several compounds of high molecular weight failed to produce fragmentation trees even with elemental composition known and were considered as "not found" (17 out of 862 in positive acquisition mode and 19 out of 291 in negative acquisition mode).

FingerID v1.4 was downloaded from https://github.com/icdishb/fingerid. The following kernels were calculated for the training and test datasets: "PPK", "CPC", "NB", "NI", "LB", "LC", "LI", "RLB", "RLI", "CP2", "CPK", "CSC"(Shen, et al., 2014). Kernel calculation was performed on HPC cluster. Kernels were combined using "ALIGN" method (Shen, et al., 2014). Parameters "sm" (m/z variance) and "si" (intensity variance) were optimized for CPK and PPK kernels using 5-fold cross-validation for the training set. Internal cross-validation was used to optimize SVM parameters. SVM models with probability estimation were constructed for fingerprint features which were set "on" in 5%-95% of the training compounds. Fingerprints were predicted using FingerID script for the test set and retrieval rates were calculated using ChemDistiller's "CSI:FingerID" scorer which relies on the pre-computed predicted fingerprints as an input for scoring candidate compounds. Generalised Jaccard similarity score was used for fingerprint matching to incorporate estimated probabilities. Results for several alternative predicted chemical formulas from SIRIUS were merged together and the total score was calculated via a multiplication of the fingerprint similarity scores and the candidate chemical formula scores reported by SIRIUS. This combination of two scores was shown to be more efficient than fingerprint similarity score on its own.

**Supplementary Note 5**. Metabolite likeness analysis

HMDB(Wishart, et al., 2013), BMDB, MassBank(Horai, et al., 2010), ChEBI (Degtyarenko, et al., 2008), PlantCyc (Zhang, et al., 2010), ECMDB (Guo, et al., 2013), and LipidMaps (Fahy, et al., 2007) were used as a "metabolite" class compound representatives. A subset of databases from ZINC (Irwin and Shoichet, 2005) (A+J Pharmtech, Acros Org, AKSci, Aldrich CPR, Amadis Chemical, Apexmol Building Blocks, Binding DB, Boroncore, Capot, Cayman, eMolecules, Fluorochem, Focus Synthesis BB, IBScreen Building Blocks, Kaironkem, Labotest, Mcule, MicroCombiChem, Molport, Oakwood Chemical, Otava Chemicals, Peakdale, PKChem, ProVence, Rare Chemicals, Ryan Scientific, Selleck Chemicals, Synblock, Synchem, SynInnova Labs BB, SynQuest Building Blocks, Synthon-Lab, UORSY and UORSY BB Make-on-demand) was used as "non-metabolite" class compound representatives. Both classes were checked for the potential overlapping entries using InChI identifiers and any overlapping entries were removed. Two classes were balanced by random sub-sampling, i.e. compounds were grouped into 1 Da wide bins according to their molecular masses and then within each bin the number of each class representatives was equalized with the larger class being randomly sub-sampled to provide identical number of compounds to the smaller class one. The total number of compounds was ~75k per class after sub-sampling. Classification was performed using SVM with radial kernel. Parameters of the SVM machine were optimized with internal 5-fold cross-validation. 94.88% 5-fold cross-validation accuracy of the model was achieved. The predictive model was applied to our set of precompiled datasets with the results summarised in **Table S4**.

**Supplementary Table 4.** Predicted percentages of metabolite-like compounds

| DataBase | Fraction of predicted metabolites, % |
| --- | --- |
| BMDB | 97.3 |
| ChEBI | 79.7 |
| DrugBank | 63.1 |
| ECMDB | 98.0 |
| FooDB | 88.9 |
| HMDB | 94.3 |
| LipidMaps | 98.9 |
| MassBank | 66.4 |
| T3DB | 61.2 |
| TestDB | 79.2 |
| YMDB | 92.0 |
| EcoCyc_MINE | 97.3 |
| KEGG_MINE | 94.4 |
| PubChem | 34.9 |
| YMDB_MINE | 98.3 |
| Zinc | 19.9 |

Metabolite likeness filter was added to ChemDistiller to optionally exclude the compounds which were predicted to be 'non-metabolites' from consideration for biological datasets. Retrieval rates with metabolite likeness filter switched on are shown in **Figure S6** for the subset of the test compounds which were classified as 'metabolites' by the model (643 out of 862 and 263 out of 291 compounds for positive and negative acquisition modes respectively).


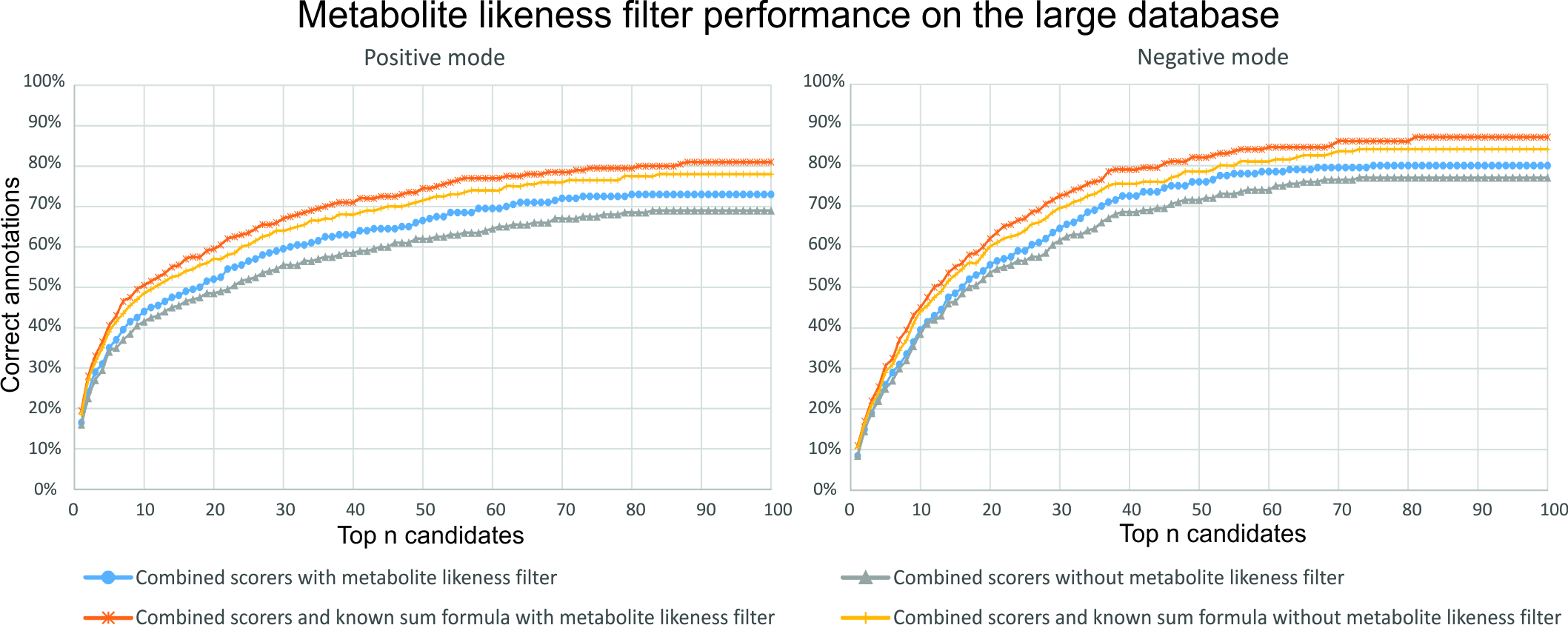


**Supplementary Figure 6.** Metabolite likeness filter performance.

**Supplementary Table 5**. Percentage overlap between returned correct results for different methods

| Positive acquisition mode | | | | | | | | |  | Negative acquisition mode | | | | | | | | | |
| --- | --- | --- | --- | --- | --- | --- | --- | --- | --- | --- | --- | --- | --- | --- | --- | --- | --- | --- | --- |
| Small database (HMDB, MassBank, ChEBI, NIST14)  Correct in **TOP 1** | | | | | | | | | | | | | | | | | | | |
|  | FingerScorer | FragScorer | FingerScorer&  FragScorer | MetFrag | | CFM-ID | | CSI:FingerID | |  |  | FingerScorer | FragScorer | FingerScorer&  FragScorer | MetFrag | CFM-ID | | | CSI:FingerID |
| FingerScorer |  | 45/56/58 | 62/71/73 | 41/48/50 | | 39/50/52 | | --/46/50 | |  | FingerScorer |  | 45/53/54 | 68/74/74 | 45/50/51 | 47/56/58 | | | --/52/54 |
| FragScorer | 45/56/58 |  | 70/75/77 | 62/62/63 | | 52/61/63 | | --/53/56 | |  | FragScorer | 45/53/54 |  | 63/68/69 | 67/66/65 | 52/56/59 | | | --/52/58 |
| FingerScorer&  FragScorer | 62/71/73 | 70/75/77 |  | 56/57/57 | | 50/59/61 | | --/50/54 | |  | FingerScorer&  FragScorer | 68/74/74 | 63/68/69 |  | 53/55/55 | 47/54/57 | | | --/55/58 |
| MetFrag | 41/48/50 | 62/62/63 | 56/57/57 |  | 53/58/59 | | --/49/51 | | |  | MetFrag | 45/50/51 | 67/66/65 | 53/55/55 |  | 53/55/56 | | | --/50/52 |
| CFM-ID | 39/50/52 | 52/61/63 | 50/59/61 | 53/58/59 |  | | --/52/55 | | |  | CFM-ID | 47/56/58 | 52/56/59 | 47/54/57 | 53/55/56 |  | | | --/52/55 |
| CSI:FingerID | --/46/50 | --/53/56 | --/50/54 | --/49/51 | --/52/55 | |  | | |  | CSI:FingerID | --/52/54 | --/52/58 | --/55/58 | --/50/52 | --/52/55 | | |  |
| Small database (HMDB, MassBank, ChEBI, NIST)  Correct in **TOP 5** | | | | | | | | | | | | | | | | | | | |
|  | FingerScorer | FragScorer | FingerScorer&  FragScorer | MetFrag | | CFM-ID | | CSI:FingerID | |  |  | FingerScorer | FragScorer | FingerScorer&  FragScorer | MetFrag | CFM-ID | | CSI:FingerID | |
| FingerScorer |  | 83/87/88 | 90/93/93 | 84/88/89 | | 80/84/84 | | --/81/81 | |  | FingerScorer |  | 77/81/81 | 87/90/91 | 78/82/83 | 74/78/79 | | --/77/78 | |
| FragScorer | 83/87/88 |  | 91/92/93 | 87/89/90 | | 82/85/86 | | --/83/83 | |  | FragScorer | 77/81/81 |  | 89/89/89 | 89/88/88 | 78/81/81 | | --/74/74 | |
| FingerScorer&  FragScorer | 90/93/93 | 91/92/93 |  | 87/90/90 | | 82/84/85 | | --/82/82 | |  | FingerScorer&  FragScorer | 87/90/91 | 89/89/89 |  | 85/86/86 | 77/80/80 | | --/77/77 | |
| MetFrag | 84/88/89 | 87/89/90 | 87/90/90 |  | | 82/85/86 | | --/81/82 | |  | MetFrag | 78/82/83 | 89/88/88 | 85/86/86 |  | 80/83/83 | | --/74/74 | |
| CFM-ID | 80/84/84 | 82/85/86 | 82/84/85 | 82/85/86 |  | | --/84/84 | | |  | CFM-ID | 74/78/79 | 78/81/81 | 77/80/80 | 80/83/83 |  | | --/75/75 | |
| CSI:FingerID | --/81/81 | --/83/83 | --/82/82 | --/81/82 | --/84/84 | |  | | |  | CSI:FingerID | --/77/78 | --/74/74 | --/77/77 | --/74/74 | --/75/75 | |  | |
| Large database (PubChem, HMDB, MassBank, ChEBI)  Correct in **TOP 20** | | | | | | | | | | | | | | | | | | | |
|  | FingerScorer | FragScorer | FingerScorer&  FragScorer | MetFrag | | CFM-ID | | CSI:FingerID | |  |  | FingerScorer | FragScorer | FingerScorer&  FragScorer | MetFrag | | CFM-ID | | CSI:FingerID |
| FingerScorer |  | 34/41/53 | 69/71/78 | 27/37/52 | | 33/41/50 | | --/44/51 | |  | FingerScorer |  | 41/50/53 | 64/67/69 | 37/45/49 | | 35/43/47 | | --/50/54 |
| FragScorer | 34/41/53 |  | 51/60/68 | 44/52/67 | | 37/49/64 | | --/46/60 | |  | FragScorer | 41/50/53 |  | 61/70/71 | 65/66/70 | | 52/58/62 | | --/50/53 |
| FingerScorer&  FragScorer | 69/71/78 | 51/60/68 |  | 36/46/59 | | 37/48/57 | | --/48/55 | |  | FingerScorer&  FragScorer | 64/67/69 | 61/70/71 |  | 50/56/59 | | 42/47/52 | | --/52/56 |
| MetFrag | 27/37/52 | 44/52/67 | 36/46/59 |  | | 27/40/57 | | --/36/52 | |  | MetFrag | 37/45/49 | 65/66/70 | 50/56/59 |  | | 50/57/63 | | --/42/47 |
| CFM-ID | 33/41/50 | 37/49/64 | 37/48/57 | 27/40/57 | |  | | --/41/55 | |  | CFM-ID | 35/43/47 | 52/58/62 | 42/47/52 | 50/57/63 | |  | | --/42/48 |
| CSI:FingerID | --/44/51 | --/46/60 | --/48/55 | --/36/52 | | --/41/55 | |  | |  | CSI:FingerID | --/50/54 | --/50/53 | --/52/56 | --/42/47 | | --/42/48 | |  |

*Note: Slash-separated percentage values are given for baseline method performance, element composition filter and formula filter added respectively.

**General Note**

Figures were prepared using MS Excel and CorelDraw. Molecular models were rendered using PyMOL (DeLano, 2008).

**Supplementary References:**

Allen, F.*, et al.* (2014) CFM-ID: a web server for annotation, spectrum prediction and metabolite identification from tandem mass spectra. *Nucleic acids research*;**42**(Web Server issue):W94-99.

Bocker, S.*, et al.* (2009) SIRIUS: decomposing isotope patterns for metabolite identification. *Bioinformatics*;**25**(2):218-224.

Chang, C.-C. and Lin, C.-J. (2011) LIBSVM: A library for support vector machines. *ACM Transactions on Intelligent Systems and Technology*;**2**(3):27:21-27:27.

Degtyarenko, K.*, et al.* (2008) ChEBI: a database and ontology for chemical entities of biological interest. *Nucleic acids research*;**36**(Database issue):D344-350.

DeLano, W.L. The PyMOL Molecular Graphics System. *DeLano Scientific LLC, Palo Alto, CA, USA* 2008.

Dührkop, K.*, et al.* (2015) Searching molecular structure databases with tandem mass spectra using CSI:FingerID. *Proc Natl Acad Sci*;**112**.

Fahy, E.*, et al.* (2007) LIPID MAPS online tools for lipid research. *Nucleic acids research*;**35**(Web Server issue):W606-612.

Guo, A.C.*, et al.* (2013) ECMDB: the *E. coli* Metabolome Database. *Nucleic acids research*;**41**(Database issue):D625-630.

Heller, S.*, et al.* (2013) InChI - the worldwide chemical structure identifier standard. *Journal of cheminformatics*;**5**(1):7.

Horai, H.*, et al.* (2010) MassBank: a public repository for sharing mass spectral data for life sciences. *Journal of mass spectrometry : JMS*;**45**(7):703-714.

Irwin, J.J. and Shoichet, B.K. (2005) ZINC - a free database of commercially available compounds for virtual screening. *J. Chem. Inf. Model*;**45**(1):177-182.

Kim, S.*, et al.* (2016) PubChem Substance and Compound databases. *Nucleic acids research*;**44**(D1):D1202-1213.

O'Boyle, N.M.*, et al.* (2011) Open Babel: An open chemical toolbox. *Journal of cheminformatics*;**3**:33.

Ruttkies, C.*, et al.* (2016) MetFrag relaunched: incorporating strategies beyond *in silico* fragmentation. *Journal of cheminformatics*;8:3.

Shen, H.*, et al.* (2014) Metabolite identification through multiple kernel learning on fragmentation trees. *Bioinformatics*;**30**(12):i157-164.

Steinbeck, C.*, et al.* (2006) Recent developments of the chemistry development kit (CDK)—an open-source java library for chemo- and bio-informatics. *Curr. Pharm. Des.*;**12**.

Weininger, D. (1988) SMILES, a chemical language and information system. 1. Introduction to methodology and encoding rules. *Journal of Chemical Information and Computer Sciences*;**28**(1):31-36.

Wishart, D.S.*, et al.* (2013) HMDB 3.0 - The Human Metabolome Database in 2013. *Nucleic acids research*;**41**(Database issue):D801-807.

Zhang, P.*, et al.* (2010) Creation of a genome-wide metabolic pathway database for Populus trichocarpa using a new approach for reconstruction and curation of metabolic pathways for plants. *Plant physiology*;**153**(4):1479-1491.

1. Department of Surgery and Cancer, Faculty of Medicine, Imperial College London, London, UK. Correspondence should be addressed to K.A.V. (kirill.veselkov04@imperial.ac.uk) [↑](#footnote-ref-2)
